# Dendritic Cell–Specific Role for Pellino2 as a Mediator of TLR9 Signaling Pathway

**DOI:** 10.4049/jimmunol.2100236

**Published:** 2021-11-01

**Authors:** Ewa Oleszycka, Aoife M. Rodgers, Linan Xu, Paul N. Moynagh

**Affiliations:** * Department of Biology, The Kathleen Lonsdale Institute for Human Health Research, Maynooth University, Maynooth, Kildare, Ireland; and; † Wellcome-Wolfson Institute for Experimental Medicine, Queen’s University Belfast, Belfast, United Kingdom

## Abstract

Ubiquitination regulates immune signaling, and multiple E3 ubiquitin ligases have been studied in the context of their role in immunity. Despite this progress, the physiological roles of the Pellino E3 ubiquitin ligases, especially Pellino2, in immune regulation remain largely unknown. Accordingly, this study aimed to elucidate the role of Pellino2 in murine dendritic cells (DCs). In this study, we reveal a critical role of Pellino2 in regulation of the proinflammatory response following TLR9 stimulation. Pellino2-deficient murine DCs show impaired secretion of IL-6 and IL-12. Loss of Pellino2 does not affect TLR9-induced activation of NF-κB or MAPKs, pathways that drive expression of IL-6 and IL-12. Furthermore, DCs from Pellino2-deficient mice show impaired production of type I IFN following endosomal TLR9 activation, and it partly mediates a feed-forward loop of IFN-β that promotes IL-12 production in DCs. We also observe that Pellino2 in murine DCs is downregulated following TLR9 stimulation, and its overexpression induces upregulation of both IFN-β and IL-12, demonstrating the sufficiency of Pellino2 in driving these responses. This suggests that Pellino2 is critical for executing TLR9 signaling, with its expression being tightly regulated to prevent excessive inflammatory response. Overall, this study highlights a (to our knowledge) novel role for Pellino2 in regulating DC functions and further supports important roles for Pellino proteins in mediating and controlling immunity.

## Key Points

- Pellino2 mediates TLR9-induced cytokine production in dendritic cells.
- Pellino2 does not play a role in TLR9 signaling in macrophages.
- Pellino2 is a limiting factor for TLR9 signaling in dendritic cells.

## Introduction

Dendritic cells (DCs) are crucial players in immunity, as they bridge innate and adaptive responses (1). DCs can be found in the tissues throughout the entire body, especially at sites where pathogen invasion can occur (2). They patrol the surrounding environment and can recognize various pathogen-associated molecular patterns using sets of different pathogen-recognition receptors (3). Among them, DNA sensors can detect nucleic acid from viruses, bacteria, and fungi (4). The TLR family member TLR9 is an endosomal receptor that recognizes unmethylated CpG motifs in pathogen DNA and provides protection against various infections (5–10) but also can detect self-DNA during pathophysiological conditions (11). Following TLR9 activation by CpG, the Toll–IL-1 resistance (TIR) domain of TLRs engages TIR domain–containing adaptor protein MyD88, which leads to activation of downstream signaling pathways, including MAPKs and NF-κB signaling cascade. Subsequent activation of the transcription factors NF-κB, the IFN-regulatory factors (IRFs), and AP-1 leads to the expression of a wide variety of genes to drive an inflammatory response (12).

Recognition of TLR ligands leads to activation of DCs that includes their maturation and upregulation of Ag presenting MHC class II (MHC II) and costimulatory molecules CD40, CD80, and CD86 and secretion of many cytokines. Depending on the nature of the pathogen recognized and specific pathway activated, DCs secrete different cytokine profiles that influence local environments and also the polarization of T cell responses that shape adaptive immunity (13). Cytokines such as TNF-α and IL-6 promote local inflammation, whereas cytokines from the IL-12 family are critical for cell-mediated immunity (14). IL-12 is biologically active as a heterodimer IL-12p70, which is composed of two subunits, IL-12p35 and IL-12p40 (15), and is essential for Th1 polarization of T cells (16). DCs produce very low levels of IL-12p70, and it has been proposed that its levels can be regulated by type I IFNs. During TLR ligand recognition, IFN-β produced by DCs feed back onto these cells and enhance IL-12 secretion (17). Thus, it highlights that DC activation can be regulated by different mechanisms at molecular and cellular levels.

Although our knowledge about DC functions is expanding, our understanding of how posttranslational modifications (PTMs) of signaling molecules can tailor activation of specific pathways and cytokine production patterns by DC is still limited (18). Ubiquitination is an important regulatory pathway for immune cell function, with E3 ubiquitin ligases playing important roles in exercising this control (19). The Pellino family of E3 ubiquitin ligases consist of three members: Pellino1, -2, and -3 (20). Early overexpression and gene knockdown studies implicated Pellino1 as a mediator in the NF-κB pathway (21), whereas Pellino2 and Pellino3 were more associated with activation of MAPK pathways (22–24). More recently, the physiological roles of the Pellino proteins are being delineated as mice deficient in each Pellino protein become available. Pellino1 is a critical mediator of immune response in various immune cells in which it targets different pathways. For instance, Pellino1 is the E3 ubiquitin ligase of RIP1 and mediates TRIF-dependent activation of NF-κB in TLR3 and TLR4 pathway but also controls activation of IFN-β promoter in macrophages (25). Pellino1 also regulates T cell activation via targeting different pathways. Pellino1 promotes ubiquitination and proteolysis of c-rel (26) and ubiquitination of TSC1, which stabilizes TSC2 and activates mTORC1 kinase (27). Thus, lack of Pellino1 has a profound effect on T cell activation, and it has been demonstrated that Pellino1 suppresses T cell–mediated autoimmunity (26), but at the same time, Pellino1 can be detrimental for T cell–mediated control of tumor growth in mice (27). Similarly, Pellino1 suppresses autoimmunity in a B cell–dependent manner as Pellino1-deficient B cells secrete autoantibodies and induce lupus-like autoimmunity in mice as Pellino1 is involved in ubiquitination and degradation of NF-κB–inducing kinase (28). Recently, several important roles of Pellino3 in immunity have been described. Pellino3 regulates TLR3 activation and type I IFN by inhibiting ubiquitination of IRF7 (29). Furthermore, Pellino3 is a key mediator of NOD2 signaling by directly catalyzing the ubiquitination of RIP2, thus triggering the expression of NOD2-responsive genes and homeostatic control of intestinal inflammation (30). Pellino3 also plays cytoprotective role, as it suppresses the proapoptotic effects of TNF by interacting with RIP1 and blocking the apoptotic caspase cascade (31). Pellino3 also controls obesity-induced expression of IL-1β and insulin resistance (32). In comparison with Pellino1 and Pellino3, there is very limited knowledge about the role of Pellino2 in immune responses. Only recently, Pellino2-deficient mice were generated, and it has been shown that Pellino2 plays a critical role in NLRP3 inflammasome activation in macrophages (33). However, no other physiological roles for Pellino2 have been described.

In this study, we explored the impact of Pellino2 in mediating DC activation and show that Pellino2 specifically targets the TLR9 signaling pathway. Pellino2 controls type I IFN and IL-12 production, which regulates T cell polarization into IFN-γ^+^ Th1 cells. Furthermore, Pellino2 expression is tightly controlled in DCs, as its overexpression leads to excessive inflammatory response. Overall, this study reveals a (to our knowledge) novel and DC-specific function of Pellino2 as a mediator of TLR9 signaling and regulator of downstream immune responses.

## Materials and Methods

### Mice

*Peli2*^−/−^ mice were generated as described before (10). Peli2-tagged mice were generated by the Transgenics Facility at Trinity Biomedical Sciences Institute, Dublin, Ireland. Two guide RNA (gRNA) targeting sequences, gRNA forward GGTCCAGTGGACTGACACCCTGG and gRNA reverse GGTGTCAGTCCACTGGACCTTGG, were designed to target the end of the coding region in exon 6 of *Peli2* gene. A repair vector was designed to remove the stop codon and insert a triple FLAG and double Strep-tag II sequence at the C terminus of *Peli2* gene. Two base changes were introduced to disrupt recutting by the Cas9. The CRISPR-RNA were assembled into Cas9 ribonucleoparticles according to standard protocols (34) and mixed with the repair vector. Generated C57BL/6J-*PELI2^em1(FLAG-Strep)Tftc^* mice were genotyped by PCR analysis of DNA isolated from ear punches using primers forward GCCAAGTACTGGTCGCAGATCC and reverse CACAGTGGTATCTGTCAGCGCC. Bone marrow from IFNAR^−/−^ mice was provided by Prof. E. Lavelle and spleens from OT-II transgenic mice were provided by Prof. K. Mills from Trinity College Dublin. Animals were housed in a specific pathogen–free animal facility and were sex and age matched for experiments.

### Reagents and Abs

LPS (ALX-581-010-L002) was from Enzo Life Sciences. Pam3CSK4; Pam2CSK4; zymosan; poly (I:C); flagellin; Clo75; Clo97; and CpG ODN 1585, 1668, and 1826; CpG ODN 1826 conjugated to FITC; poly dA:dT; VACV70; and cGAMP were from InvivoGen. Lipofectamine 2000 was from Thermo Fisher Scientific. *N*-[1-(2,3-dioleoyloxy)propyl]-*N,N,N*-trimethylammonium methyl-sulfate (DOTAP) was from Merck. Murine rIFN-β (12405) was from PBL Assay Science. PMA (P8139), ionomycin (I0634), and brefeldin A (B6542) were from Sigma-Aldrich. All Abs were used at a dilution of 1:1000 unless otherwise stated. Anti–p-IκBα (9246), anti–p-ERK (9101), anti-ERK (9102), anti-IRF1 (8478), anti-IRF3 (4302), anti-IRF7 (72073), anti–p-p38 (9211), anti-p38 (9212), anti–p-Jnk (9251), anti-Jnk (9252), anti-myc (2276), anti–p-STAT1 (7649), and anti-STAT1 (9172) were from Cell Signaling Technology; anti–IκB-α (C-21; sc-371) and anti-GFP (FL; sc-8334) were from Santa Cruz Biotechnology; anti–β-actin (AC-15; A 1978) (1:5000) was from Sigma-Aldrich; anti–Strep-tag II (ab76949) was from Abcam; anti-mouse IRDye 680 (926-68070) (1:5000) and anti-rabbit IRDye 800 (926-32211) (1:5000) were from LI-COR Biosciences; and anti-mouse HRP was from Promega. Anti-CD16/CD32 (2.4G) was from BD Pharmingen. Zombie NIR Fixable Dye (1:200), anti-CD4 (RM4-5) (1:200), IFNAR blocking Ab (clone MAR1-5A3), and isotype control Ab (clone MOPC-21) were from BioLegend. Anti-CD80 (16-10A1) (1:200), anti-CD86 (GL1) (1:200), anti-CD40 (1C10) (1:200), anti-CD11c (N418) (1:200), anti–MHC II (M5/114.15.2), and anti–IFN-γ (XMG1.2) (1:200) were from eBioscience.

### Cell culture

HEK293T (ATCC CRL-11268) were cultured in RPMI 1640 GlutaMAX medium (Life Technologies) supplemented with 10% (v/v) FBS (Sigma-Aldrich), 100 U/ml penicillin, and 100 μg/ml streptomycin (Life Technologies). Bone marrow–derived DCs (BMDCs) and bone marrow–derived macrophages (BMDMs) were generated from bone marrow isolated from tibiae and femurs of mice. BMDCs were grown in RPMI 1640 plus GlutaMAX supplemented with 10% (v/v) FBS, 100 U/ml penicillin, and 100 μg/ml streptomycin and 20 ng/ml of GM-CSF (PeproTech). On day 9–10, cells were harvested and plated for experiments. BMDMs were grown in DMEM (HyClone) supplemented with 10% (v/v) FBS, 100 U/ml penicillin, and 100 μg/ml streptomycin and 20% L929 conditioned medium containing M-CSF. On day 5–6, adherent cells were harvested and plated for experiments. Splenic CD11c^+^ DCs were isolated using CD11c MicroBeads UltraPure mouse kit from Miltenyi Biotec according to manufacturer’s instructions. Cell purity was >90%.

### Staphylococcus aureus *infection*

*Staphylococcus aureus* JE2 (CA-MRSA USA300; kindly provided by Prof. S. Foster, University of Sheffield) was cultured in 20 ml of brain heart infusion broth (BHI; Oxoid) with aeration at 220 rpm or on BHI agar at 37°C. *S. aureus* and streaked from frozen stocks on BHI agar plates and incubated overnight at 37°C. From these plates, overnight cultures were prepared by inoculation of 20 ml BHI with a colony of *S. aureus.* Following overnight incubation, a subculture with an OD of 600 nm at 0.05 was prepared in BHI and grown for 2.5 h. Bacterial suspensions were subsequently prepared in PBS, and the concentrations were estimated by measuring the absorbance at OD of 600 nm. BMDC were cultured and plated in antibiotic-free media and were infected with *S. aureus* for 2 h, after which media was replaced with fresh media containing gentamicin (200 μg/ml) to kill extracellular bacteria. Cytokine levels were quantified 24 h later by ELISA.

### T cell responses

BMDCs were stimulated with OVA alone or TLR ligands with OVA for 18 h before the addition of magnetic bead (Miltenyi Biotec)–sorted CD4 OT-II T cells (ratio 5:1 T cell/DC). T cell responses were analyzed 72 h later. Supernatants were removed and analyzed by ELISA. Cells were treated with 100 ng/ml PMA (Sigma-Aldrich), 500 ng/ml ionomycin (Sigma-Aldrich), and 10 µg/ml brefeldin A (Sigma-Aldrich) for 5 h. Cells were washed, stained, and analyzed by flow cytometry.

### Lentivirus transduction

The plasmids myc-tagged Pellino2, Pellino2-C334A/C337A (Peli2-RING), and Pellino2-R106A/S136A (Peli2-FHA) were generated in-house and subcloned into lentiviral pLV-CAG-IRES-GFP vector (plasmid no. 69047; Addgene). The empty pLV vector (as control) or pLV containing the indicated Pellino2 construct (10 μg) was cotransfected with the psPAX2 (Addgene plasmid no. 12260) (12 μg) and pMD2.G envelope vector (Addgene plasmid no. 12259) (6.3 μg) into a T175 cm^2^ flask of HEK293T cells in RPMI 1640 plus GlutaMAX supplemented with 10% (v/v) FBS, 100 U/ml penicillin, and 100 μg/ml streptomycin (25 ml). Media was refreshed after 16 h. After 48 h, the lentivirus-containing medium was collected. BMDCs were transduced on day 6 cell culture with virus containing medium (6 ml) in T175 cm^2^ flasks for 1 h, and 24 ml media was added to cells to allow culturing. BMDMs were transduced on day 3 cell culture with virus containing medium (5 ml) in T175 cm^2^ flasks for 1 h, and 20 ml media was added to cells to allow culturing. Lentivirus-infected cells were incubated at 37°C until day of plating and then used for experiments. For some experiments, lentivirus-infected BMDCs were sorted to obtain only transduced BMDCs (GFP^+^). GFP^+^ BMDCs were purified by flow cytometry using an FACSAria Fusion (BD Biosciences). Purity was at least 95%.

### Flow cytometry

Cells were washed with PBS, pelleted by centrifugation (400 × *g* for 5 min at 4°C) and stained with Zombie NIR Fixable Dye for 30 min in the dark and on ice. After washing with PBS, cells were incubated with 50 μl of FACS buffer (2% FBS in PBS) mixed with anti-CD16/CD32 monoclonal Abs. Cells were then stained with the fluorochrome-labeled Abs for 15 min in the dark and on ice. Cells were washed twice and resuspended in 100 μl of FACS buffer and fixed using 100 μl Intracellular Fixation Buffer (Thermo Fisher Scientific). For intracellular staining, cells were permeabilized and stained using 200 μl 1× Permeabilization Buffer prepared from 10× Permeabilization Buffer (Thermo Fisher Scientific). Cells were washed twice using 1× Permeabilization Buffer and resuspended in 100 μl of FACS buffer. All compensations were set up using OneComp beads (eBioscience), or if not applicable, cells were stained with a dye. Samples were acquired on BD FACSCanto II or Attune NxT Acoustic Focusing Cytometer using FACSDiva (BD Biosciences) or Attune NxT software (Thermo Fisher Scientific), and the data were analyzed using FlowJo software (Tree Star).

### Western blotting

Primary BMDCs were cultured in 12-well plates (1 × 10^6^ cells/ml; 1 ml). For whole-cell lysate analysis, cells were lysed using 2 × SDS-PAGE sample buffer and heated to 95°C for 5 min. Samples were resolved by SDS-PAGE, transferred to nitrocellulose membranes, and analyzed by immunoblot with appropriate Abs. Immunoreactivity was visualized by the Odyssey Imaging System (LI-COR Biosciences) or ECL.

### Real-time PCR

Primary BMDCs were cultured in 12-well plates (1 × 10^6^ cells/ml; 1 ml). Total RNA was extracted from tissues or cells using TRIzol (Invitrogen). cDNA was generated from 200–400 ng RNA using qScript cDNA Synthesis Kit (Quantabio), and real-time PCR analyses were performed with DreamTaq Green PCR Master Mix (Thermo Fisher Scientific) using an Applied Biosystems StepOnePlus Real-Time PCR System according to the manufacturer’s instructions. The abundance of each mRNA was normalized relative to PCR of the housekeeping gene hypoxanthine-guanine phosphoribosyltransferase (Hprt) from the corresponding sample (Ct_Gene_ – Ct_HPRT_ = ΔCt). Furthermore, ΔCt from control samples were averaged and were subtracted from the ΔCt of each sample (ΔCt_1_ − ΔCt_ctrl mean_ = ΔΔCt_1_). Fold induction was calculated as 2^(−ΔΔCt)^. The following were used: mouse HPRT, forward AGGGATTTGAATCACGTTTG and reverse TTTACTGGCAACATCAACAG; mouse *Peli2*, forward AGGGTAAAGGATACACTCAC and reverse TCTTAGAAGACAATCCCAGG; mouse *Il6*, forward AAGAAATGATGGATGCTACC and reverse GAGTTTCTGTATCTCTCTGAAG; mouse *Il12a*, forward AAGATGTACCAGACAGAGTTC and reverse ATGCAGAGCTTCATTTTCAC; mouse *Il12b*, forward CATCAGGGACATCATCAAAC and reverse CTCTGTCTCCTTCATCTTTTC; mouse *Ifnb*, forward AACTTCCAAAACTGAAGACC and reverse AACTCTGTTTTCCTTTGACC; mouse *Cxcl10*, forward GACGGTCCGCTGCAACTG and reverse GCTTCCCTATGGCCCTCATT; mouse *Isg20*, forward ACGGACTACAGAACCCAAGTCAGC and reverse ACCACCAGCTTGCCTTTCAGAAG; and mouse *Oas1a*, forward TCCCAGAATCTATGCCATCC and reverse TTCCCCAGCTTCTCCTTACA.

### Measurement of cytokine levels by ELISA

Concentrations of TNF-α, IL-6, IL-12p40, and IL-12p70 were measured using kits from R&D Systems. IFN-β was detected using coating Ab for IFN-β (7F-D3) (1:1000) from Santa Cruz Biotechnology, detection polyclonal IFN-β Ab (32400-1) from PBL, and anti-rabbit Ab conjugated with HRP (W401B) from Promega.

### Densitometry analysis

Densitometry analysis was performed using ImageJ software.

### Statistical analysis

For in vitro studies, unpaired two-tailed *t* test was used when only two groups were compared; when multiple groups were compared, two-way ANOVA was used; when significant differences were found, the Sidak multiple comparisons test was used to identify differences between groups.

## Results

### Pellino2 mediates proinflammatory cytokine production in response to CpG in DCs

**FIGURE 1.**
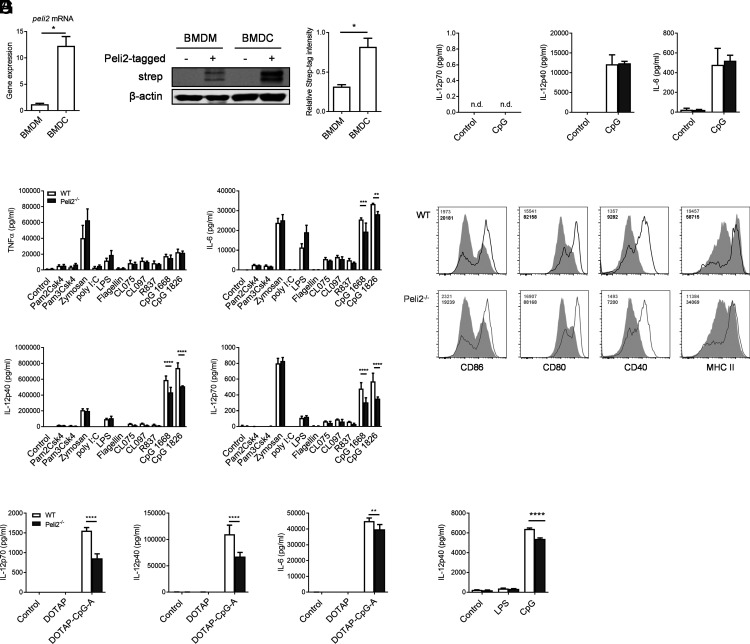
Pellino2 is required for proinflammatory cytokine production in response to CpG in DCs. (**A**) mRNA level of *Peli2* in WT BMDMs and BMDCs. (**B**) Protein level of strep-tagged Pellino2 protein in BMDMs and BMDCs generated from WT and Peli2-tagged mice. (**C**) ELISA of TNF-α, IL-6, IL-12p40, and IL-12p70 secreted in medium from BMDCs isolated from WT and Pellino2-deficient (*Peli2*^−/−^) mice and treated with 50 ng/ml Pam2CSK, 50 ng/ml Pam3CSK, 10 μg/ml zymosan, 50 μg/ml poly(I:C), 5 ng/ml LPS, 1 μg/ml flagellin, 1 μg/ml Clo75, 1 μg/ml Clo97, 5 μg/ml R837, and 1 μg/ml CpG ODN 1668 or 1826 for 24 h. (**D**) ELISA of IL-12p70, IL-12p40, and IL-6 secreted in medium from WT and *Peli2*^−/−^ BMDCs treated with 5 μg/ml DOTAP alone or 5 μg/ml DOTAP with 5 μg/ml CpG ODN 1585 for 24 h. (**E**) ELISA of IL-12p40 secreted in medium from enriched splenic WT and *Peli2*^−/−^ CD11c^+^ DCs treated with 5 ng/ml LPS or 10 µg/ml CpG ODN 1826 for 24 h. (**F**) ELISA of IL-12p70, IL-12p40, and IL-6 secreted in medium from WT and *Peli2*^−/−^ BMDMs treated with 1 μg/ml CpG ODN 1826 for 24 h. (**G**) WT and *Peli2*^−/−^ BMDCs were incubated with CpG ODN 1826 for 24 h, and expression of surface CD86, CD80, CD40, and MHC II was analyzed by flow cytometry. (A) Results show mean of two independent experiments. Data show means + SEM. (B) Immunoblots show representative of three independent experiments. Data show means + SEM of three independent experiments. (C and F) Results are representative of two independent experiments. Data show means + SD for three technical replicates. (D, E, and G) Results are representative of three independent experiments. Data show means + SD for three technical replicates. WT BMDC versus *Peli2*^−/−^ BMDC. *p < 0.05, **p < 0.01, ***p < 0.001, ****p < 0.0001, (A and B) unpaired *t* test and (C–E) two-way ANOVA. n.d., not detected.

We recently highlighted the physiological role of Pellino2 in the NLRP3 signaling pathway in macrophages. BMDMs from these mice show normal induction of proinflammatory cytokines in response to various TLR ligands. However, Pellino2 deficiency resulted in significant impairment of NLRP3 inflammasome activation and IL-1β secretion (33). However, little is known about the role of Pellino2 in other immune cells. In this study, we focused on the potential of Pellino2 to control DC responses, as it has been reported that DCs express higher levels of *Peli2* in comparison with macrophages (26). To confirm this, we compared expression of *Peli2* in BMDMs and BMDCs from wild-type (WT) mice using RT-PCR analysis. BMDCs express higher levels of *Peli2* mRNA than BMDMs (Fig. 1A). To date, there is no report showing protein levels of Pellino2 in different cells and tissues. We have assessed a number of commercially available Abs that have been purported to recognize Pellino2, but we have been unable to show any immunoreactivity with Pellino2. To overcome this limitation, we generated transgenic mice that express Pellino2 with additional FLAG and Strep-tag II (Supplemental Fig. 1). Using these transgenic mice, Peli2-tagged BMDMs and BMDCs were generated, and expression levels of Pellino2 were assessed using an anti–Strep-tag II Ab. Similar to mRNA analysis, Pellino2 protein expression was higher in BMDCs than in BMDMs (Fig. 1B). To explore the role of Pellino2 in DCs, we initially compared the response of WT BMDCs with *Peli2*^−/−^ BMDCs to a wide array of TLR ligands and measured induction of proinflammatory cytokines: TNF-α, IL-6, IL-12p40, and IL-12p70. Pam2Csk and Pam3Csk (TLR1/2 ligands), zymosan (TLR2/6 ligand), poly I:C (TLR3 ligand), LPS (TLR4 ligand), flagellin (TLR5 ligand), R837 (TLR7 ligand), and CL075 and CL097 (TLR7/8 ligands) induced comparable levels of these cytokines in WT and *Peli2*^−/−^ BMDCs. However, the levels of IL-6, IL-12p40, and IL-12p70 were reduced in Pellino2-deficient BMDCs treated with CpG type B ODN 1668 and 1826 (TLR9 ligands) relative to similarly treated WT BMDCs (Fig. 1C). Next, we analyzed whether Pellino2 controls TLR9 activation with the type A CpG ODN 1585. We transfected WT and *Peli2*^−/−^ BMDCs with CpG ODN 1585 and analyzed cytokine secretion. Similar to studies using CpG type B, *Peli2*^−/−^ BMDCs secreted reduced levels of IL-12p40, IL-12p70, and IL-6 relative to WT BMDCs when treated with CpG type A (Fig. 1D). Having established that Pellino2 mediates cytokine response in BMDCs, we next assessed if Pellino2 plays a similar mediatory role in primary dendritic directly isolated from tissue. To this end, we isolated splenic CD11c^+^ DCs and demonstrated that Peli2^−/−^ splenic CD11c^+^ DCs secreted reduced IL-12p40 when treated with CpG (Fig. 1E) relative to similarly treated WT splenic CD11c^+^ DCs. These data suggest a selective role for Pellino2 in controlling the TLR9 signaling pathway in DCs. Interestingly, in a previous report, we did not detect any differences in TLR9 signaling in WT and *Peli2*^−/−^ macrophages, as evidenced by CpG inducing comparable levels of TNF-α, IL-6, CXCL1, or RANTES secretion in WT and *Peli2*^−/−^ cells (33). To further investigate the potential role of Pellino2 in TLR9 signaling in macrophages, we measured the production of IL-6, IL-12p40, and IL-12p70 in WT and *Peli2*^−/−^ BMDMs stimulated with CpG. In contrast to BMDCs, WT and *Peli2*^−/−^ BMDMs secreted comparable levels of IL-12p40 and IL-6 in response to CpG, whereas IL-12p70 was not detected (Fig. 1F). These data highlight that Pellino2 plays various roles in different immune cells with DC-specific function in the TLR9 signaling pathway.

We next examined if Pellino2 mediates the expression of all TLR9-responsive genes in DCs or if its role was restricted to gene subsets and specific functions. We thus examined the ability of CpG to modulate surface expression of MHC II and the costimulatory molecules CD40, CD80, and CD86 in WT and *Peli2*^−/−^ BMDCs. WT and *Peli2*^−/−^ BMDCs were similarly responsive to CpG in inducing the expression of these membrane proteins (Fig. 1G). These data suggest that Pellino2 mediates the expression of a subset of TLR9-responsive genes in DCs with important roles in mediating production of proinflammatory cytokines, whereas the overall activation of DCs appears to be independent of Pellino2, at least based on analysis of surface protein markers for DC activation.

### Pellino2 mediates transcriptional upregulation of TLR9-responsive genes in DCs but does not modulate NF-κB and MAPK signaling in response to CpG

**FIGURE 2.**
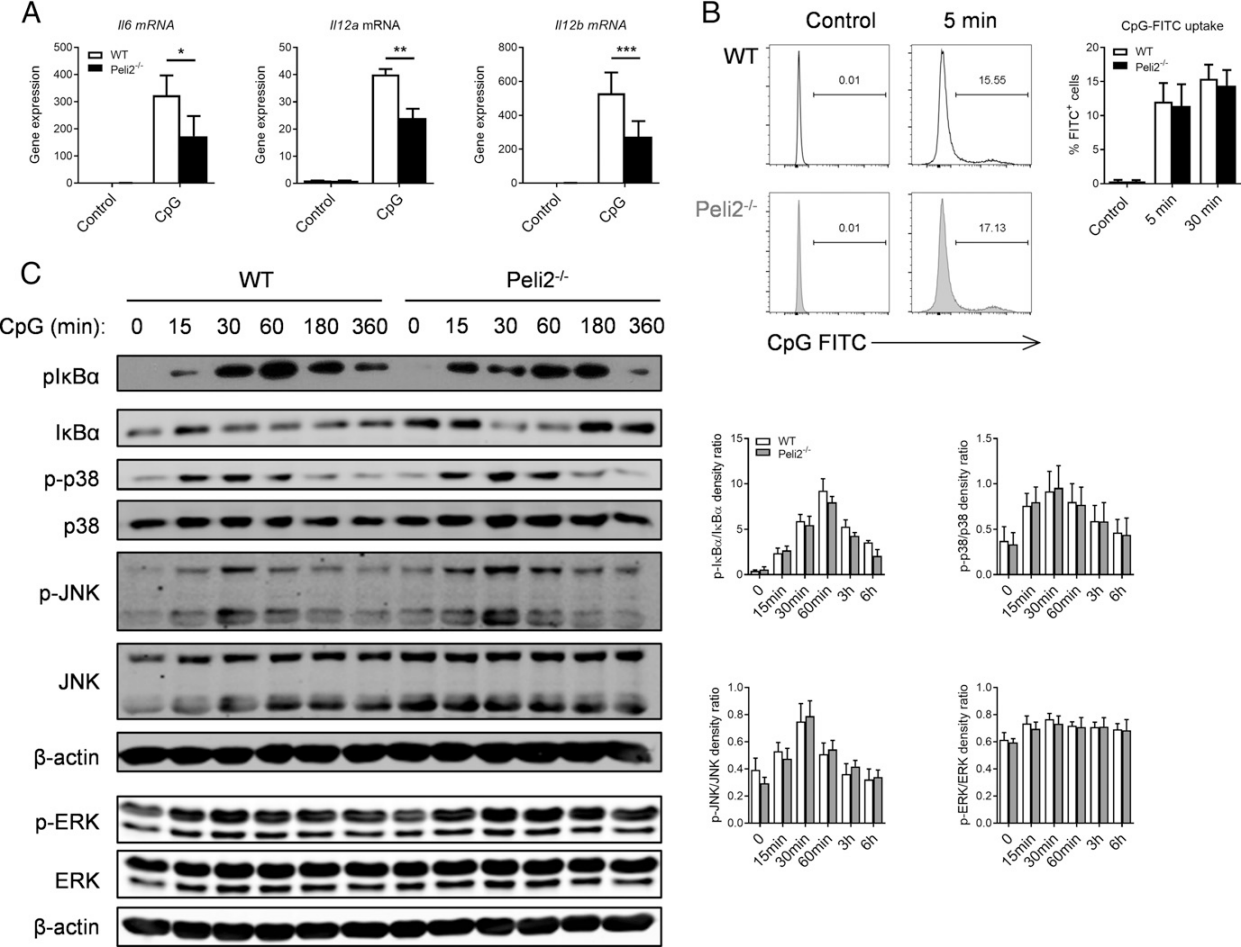
Pellino2 mediates CpG-induced transcription of genes encoding proinflammatory cytokines in DCs in a manner independent of NF-κB and MAPKs pathways. (**A**) WT and *Peli2*^−/−^ BMDCs were incubated with CpG ODN 1826, and the expression of *Il6*, *Il12a*, and *Il12b* mRNA was determined in cells after 3 h by RT-PCR. (**B**) WT and *Peli2*^−/−^ BMDCs were incubated with 1 µg/ml CpG ODN 1826 conjugated with FITC and CpG uptake and assessed by flow cytometry at indicated time points. (**C**) Immunoblot and densitometry analysis of p–IκB-α, total IκB, p-p38, total p38, p-JNK, total JNK, p-ERK, and ERK in lysates of WT and *Peli2*^−/−^ BMDCs stimulated with 1 µg/ml CpG ODN 1826 for indicated durations. β-actin was used as a loading control. (A) Results show mean of three independent experiments. Data show means + SEM. (B) Flow cytometry histograms are representative of three independent experiments. Data presented in the graph show mean of three independent experiments. Data show means + SEM. (C) Immunoblots show representative of three independent experiments. Data show means + SEM of three independent experiments. WT BMDC versus *Peli2*^−/−^ BMDC. * *p* < 0.05, **p < 0.01, ***p < 0.001, two-way ANOVA.

We next explored the mechanistic basis to the regulatory effects of Pellino2 on proinflammatory protein expression in DCs by exploring if loss of Pellino2 affects the ability of CpG to induce transcriptional upregulation of *Il-6* and *Il12* mRNA. WT and *Peli2*^−/−^ BMDCs were treated with CpG and measured for levels of mRNA by quantitative RT-PCR. Similar to secretion levels of encoded cytokines, Pellino2 deficiency resulted in decreased CpG-induced mRNA levels of *Il6*, *Il12a*, and *Il12b* in BMDCs (Fig. 2A), suggesting that Pellino2 may mediate early signaling events in the TLR9 pathway. We thus explored the role of Pellino2 in mediating signaling events that are proximal to TLR9.

The activation of TLR9 and subsequent cytokine production requires CpG internalization by DCs (35). To exclude the possibility that there is any defect with CpG uptake in Pellino2-deficient cells leading to decreases in cytokine production, WT and Pellino2-deficient DCs were incubated with CpG conjugated with FITC and CpG uptake and measured by flow cytometry. CpG was internalized as quickly as 5 min, and there was no difference between WT and Pellino2-deficient BMDCs in relation to the efficiency of uptake of CpG (Fig. 2B).

We next examined the role of Pellino2 in the intracellular signaling pathways downstream of TLR9 and that drive the expression of proinflammatory cytokines. To this end, we analyzed the activation of the NF-κB and MAPK pathways in response to CpG stimulation of WT and Pellino2-deficient BMDCs. CpG promoted time-dependent activation of NF-κB and MAPK pathways, as measured by phosphorylation of the NF-κB inhibitory protein IκBα and p38 MAPK, JNK, and ERK, with similar patterns of activation in WT and *Peli2*^−/−^ BMDCs (Fig. 2C). These findings indicate that Pellino2 mediates TLR9-induced expression of the proinflammatory cytokines IL-12 and IL-6 but in a manner independent of the NF-κB and MAPK pathways.

### Pellino2 mediates TLR9-induced expression of type I IFN in DCs

**FIGURE 3.**
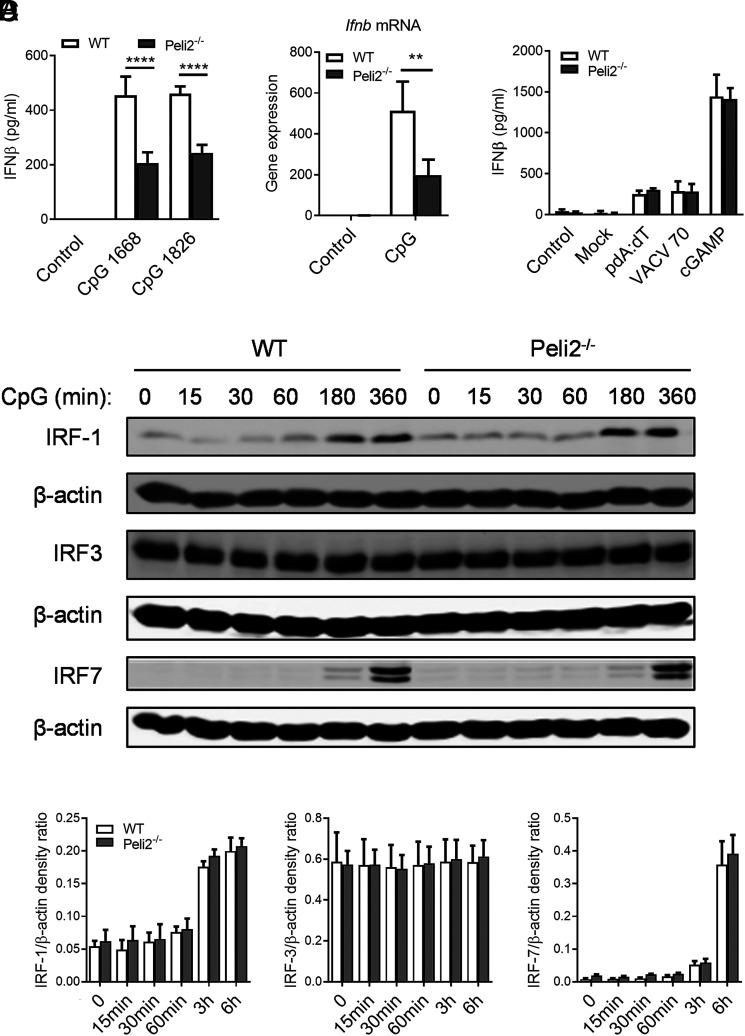
Pellino2 is required for IFN-β secretion in response to CpG in DCs. (**A**) WT and *Peli2*^−/−^ BMDCs were treated with 1 μg/ml CpG ODN 1826. After 24 h, supernatants were tested for IFN-β by ELISA. (**B**) WT and *Peli2*^−/−^ BMDCs were treated with 1 μg/ml CpG ODN 1826. After 3 h, *Ifnb* mRNA levels were determined in cells by RT-PCR. (**C**) WT and *Peli2*^−/−^ BMDCs were treated with lipofectamine alone and transfected with 10 µg/ml polydA:dT and 10 µg/ml VACV70 or 10 µg/ml cGAMP alone. After 24 h, supernatants were tested for IFN-β by ELISA. (**D**) Immunoblot and densitometry analysis of total IRF1, IRF3, and IRF7 in lysates of WT and *Peli2*^−/−^ BMDCs stimulated with 1 µg/ml CpG ODN 1826 for indicated durations. β-actin was used as a loading control. (A and C) Results are representative of three independent experiments. Data show means + SD for three technical replicates. (B) Results show mean of three independent experiments. Data show means + SEM. (D) Immunoblots show representative of three independent experiments. Data show means + SEM of three independent experiments. WT BMDC versus *Peli2*^−/−^ BMDC. **p < 0.01, ****p < 0.0001, two-way ANOVA.

IL-12 secretion has been shown to be increased by type I IFN in DCs following TLR9 activation (17). Therefore, we analyzed whether Pellino2 might mediate not only expression of proinflammatory cytokines IL-12 and IL-6 but also IFN-β. WT and *Peli2*^−/−^ BMDCs were thus stimulated with CpG, and expression of type I IFN was measured. *Peli2*^−/−^ BMDCs secreted lower amounts of IFN-β in response to CpG compared with WT cells (Fig. 3A). In addition, *Peli2*^−/−^ BMDCs expressed lower levels of *Ifnb* mRNA when compared with WT BMDCs (Fig. 3B). Again, this role for Pellino2 appears to be specific to the TLR9 pathway because WT and *Peli2*^−/−^ BMDCs showed comparable levels of IFN-β when stimulated by ligands for other DNA sensing receptors (poly dA:dT, ligand of multiple DNA sensors; VACV70, ligand of IFI16; and cGAMP, STING agonist) (Fig. 3C).

IRFs control type I IFN induction, and it has been shown that IRF1 and IRF7 mediate TLR9 responses in DCs (36–38), whereas IRF3 is necessary for TLR4 and cytoplasmic DNA detection (39). Therefore, to assess whether Pellino2 mediates IFN-β expression via IRFs, we analyzed their activation in WT and *Peli2*^−/−^ BMDCs. Activation of IRF1 and IRF7 was comparable in these cells, whereas IRF3 levels were not altered in WT and *Peli2*^−/−^ BMDCs stimulated with CpG (Fig. 3D).

**FIGURE 4.**
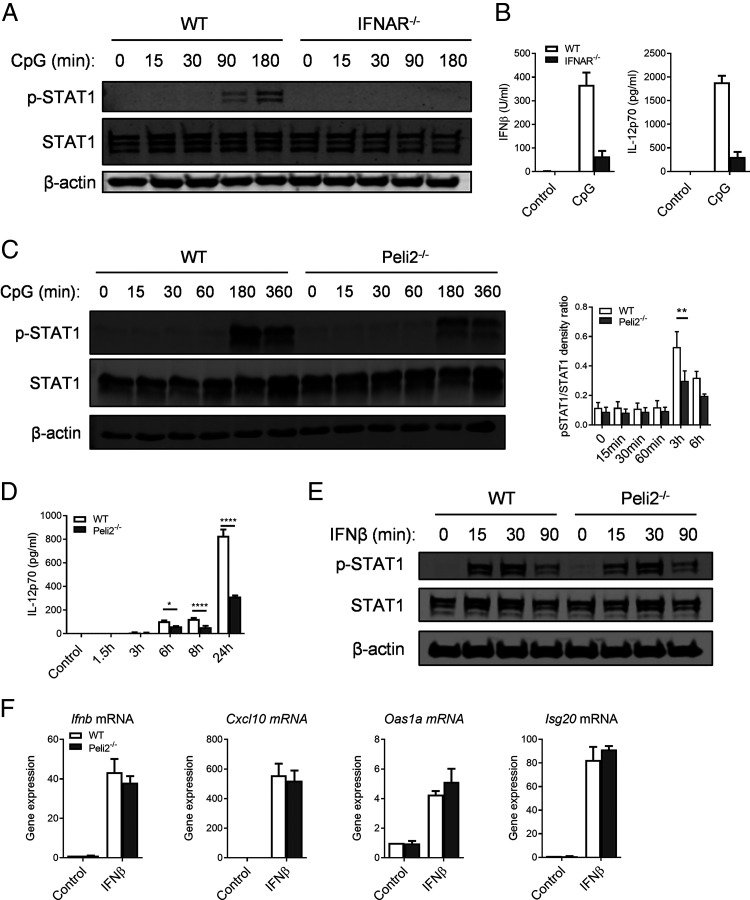
Pellino2 is required for STAT1 activation in DCs following TLR9 stimulation. (**A**) WT and IFNAR^−/−^ BMDCs were treated with 1 μg/ml CpG ODN 1826. Immunoblot analysis of p-STAT1, STAT1, and β-actin was performed for indicated times. (**B**) Cell culture supernatants were tested for IFN-β and IL-12p70 after 24 h by ELISA. (**C**) Immunoblot and densitometry analysis of p-STAT1, STAT1 in lysates of WT, and *Peli2*^−/−^ BMDCs stimulated with 1 µg/ml CpG ODN 1826 for indicated times. β-actin was used as a loading control. (**D**) ELISA of IL-12p70 secreted in medium from WT and *Peli2*^−/−^ BMDCs treated with 1 μg/ml CpG ODN 1826 for indicated time points. (**E**) Immunoblot analysis of p-STAT1 and STAT1 in lysates of WT and *Peli2*^−/−^ BMDCs stimulated with 500 U rIFN-β for indicated durations. β-actin was used as a loading control. (**F**) WT and *Peli2*^−/−^ BMDCs were incubated with rIFN-β, and the expression of *Ifnb*, *Cxcl10*, *Oas1a*, and *Isg20* mRNA was determined in cells after 3 h by RT-PCR. (A) Immunoblotting results are representative of three independent experiments. (B and D) ELISA data represent one of three independent experiments. Data show means + SD. (C) Results are representative of four independent experiments. Data show means + SEM of four independent experiments. (E) Data are representative of three independent experiments. (F) Data show mean of three independent experiments. Data show means + SEM. WT BMDC versus *Peli2*^−/−^ BMDC. *p < 0.05, **p < 0.01, ****p < 0.0001, two-way ANOVA.

Because TLR9 signaling can induce IFN-β in DCs and feed forward onto these cells to further enhance IL-12 secretion (40), we analyzed whether reduced secretion of IL-12p70 in *Peli2*^−/−^ BMDCs could be linked to decreased IFN-β secretion. First, we employed BMDCs that were generated from IFNAR^−/−^ mice. IFNAR^−/−^ BMDCs failed to show CpG-induced phosphorylation of the transcription factor STAT1 that is normally activated downstream of IFNAR (Fig. 4A). Notably, IFNAR^−/−^ BMDCs also showed reduced levels of IFN-β and IL-12 in response to CpG demonstrating that CpG induces IFN-β to feed forward and further enhance production of IFN-β and IL-12 (Fig. 4B). This is consistent with reduced IFN-β production in Pellino2-deficient BMDCs that could be associated with reduced feed-forward expression of IL12p70. To further confirm that Pellino2 regulates this feed-forward mechanism, we measured CpG-induced activation of STAT1 in WT and Pellino2-deficient cells. CpG induced time-dependent phosphorylation of STAT1 in WT BMDC, and this was strongly reduced in *Peli2*^−/−^ BMDCs (Fig. 4C). Next, to further analyze the relationship between type I IFN and IL-12, we analyzed whether p-STAT1 activation proceeds IL-12p70 secretion. Indeed, although the peak of p-STAT1 can be detected at 3 h poststimulation, both WT and Peli2^−/−^ BMDCs produced IL-12p70 from 6 h poststimulation. Moreover, *Peli2*^−/−^ BMDCs secreted lower levels of IL-12p70 as early as 6 h poststimulation (Fig. 4D). We next evaluated whether Pellino2 might play a role in the initial induction of IFN-β production or subsequent feed forward by IFN-β via IFNAR signaling. To this end, we characterized signaling in response to exogenous IFN-β in WT and *Peli2*^−/−^ BMDCs. Pellino2 deficiency did not affect the ability of IFN-β to induce phosphorylation of STAT1 (Fig. 4E). Furthermore, the expression of IFN-responsive genes such as *Ifnb*, *Cxcl10*, *Oas1a*, and *Isg20* was comparable in WT and *Peli2*^−/−^ BMDCs (Fig. 4F). These data confirm that the primary role of Pellino2 is as a mediator of IFN-β induction and does not play a direct role in the type I IFN secondary response.

**FIGURE 5.**
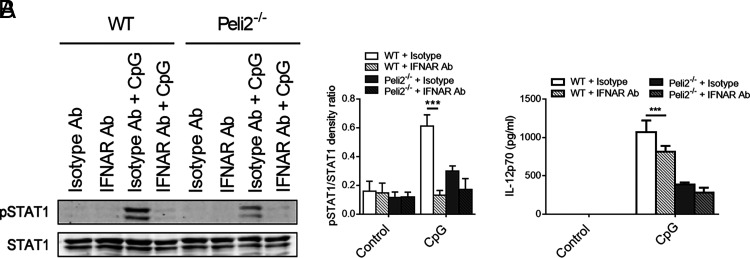
Pellino2 promotes proinflammatory cytokine secretion by type I IFN feed-forward loop. (**A**) Immunoblot analysis of p-STAT1, STAT1 in lysates of WT, and *Peli2*^−/−^ BMDCs treated with 10 µg/ml of isotype control Ab or IFNAR blocking Ab and stimulated with 1 µg/ml CpG ODN 1826 for 3 h. (**B**) ELISA of IL-12p70 secreted in medium from WT and *Peli2*^−/−^ BMDCs treated with 10 µg/ml of isotype control Ab or IFNAR blocking Ab and stimulated 1 μg/ml CpG ODN 1826 for 24 h. (A and B) Data are representative of three independent experiments. Data show means + SD. WT BMDC versus *Peli2*^−/−^ BMDC. ***p < 0.001, two-way ANOVA.

Next, to analyze the link between IFN-β and IL-12p70, we analyzed cytokine expression and production in the presence of an IFNAR blocking Ab. First, we confirmed that blocking IFNAR significantly reduced phosphorylation of STAT1 in WT and *Peli2*^−/−^ BMDCs treated with CpG (Fig. 5A). Next, we analyzed whether blocking IFNAR would affect IL-12p70 secretion by WT and Peli2^−/−^ BMDCs stimulated with CpG. WT BMDCs treated with IFNAR blocking Ab secreted less IL-12p70 when compared with WT BMDCs treated with isotype control Ab (Fig. 5B). Although Peli2^−/−^ BMDCs treated with isotype control Ab showed strongly reduced induction of IL-12p70 by CpG relative to similarly treated WT BMDCs, the IFNAR blocking Ab failed to further suppress levels of IL-12p70 in CpG-stimulated Peli2^−/−^ BMDCs, suggesting that Pellino2 may be mediating at least some of its effects on IL-12p70 by its direct role in the initial induction of IFN-β.

### Pellino2 is a limiting factor for TLR9 signaling in DCs

**FIGURE 6.**
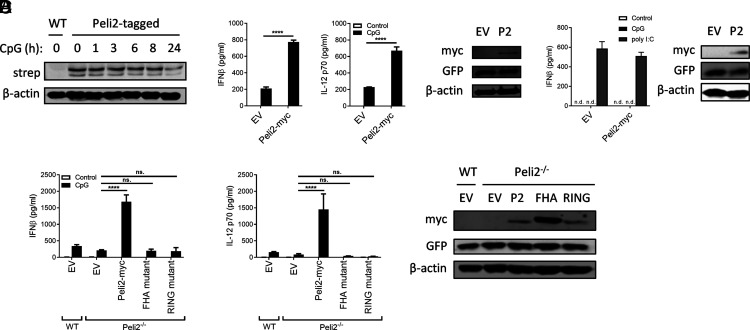
Enhanced expression of Pellino2 in DCs augments TLR9-induced expression of IFN-β. (**A**) Immunoblot analysis of Strep-tag II in WT and Peli2-tagged BMDCs treated with 1 µg/ml CpG ODN 1826 for indicated times. β-actin was used as a loading control. (**B**) WT BMDCs were infected with empty lentivirus (EV) as control or with lentivirus containing an expression construct encoding myc-tagged murine Pellino2. Cells were treated with 1 µg/ml CpG ODN 1826, and supernatants were analyzed for IFN-β and IL-12p70 production after 24 h. Immunoblot analysis of myc, GFP, and β-actin was performed on EV, and Peli2-myc lentivirus transduced WT BMDCs. (**C**) WT BMDMs were infected with EV as control or with lentivirus containing an expression construct encoding myc-tagged murine Pellino2. Cells were treated with 10 µg/ml CpG ODN 1826 or 25 µg/ml poly I:C, and supernatants were analyzed for IFN-β production after 24 h. Immunoblot analysis of myc, GFP, and β-actin was performed on EV, and Peli2-myc lentivirus transduced WT BMDMs. (**D**) WT and *Peli2*^−/−^ BMDCs were infected with EV as control and *Peli2*^−/−^ BMDCs with lentivirus containing an expression construct encoding myc-tagged murine Pellino2, RING mutant (Peli2-RING), or Pellino2 FHA mutant (Peli2-FHA). GFP^+^-sorted BMDCs were treated with 1 µg/ml CpG ODN 1826, and supernatants were analyzed for IFN-β and IL-12p70 production after 24 h. Immunoblot analysis of myc, GFP, and β-actin was performed on sorted BMDCs. EV versus Peli2-myc or mutant Peli2. Results are representative of three independent experiments. Data show means + SD for three technical replicates. ****p < 0.0001, two-way ANOVA. n.d., not detected; ns, not significant.

Given the physiological role for Pellino2 in the TLR9 pathway, we were keen to characterize the potential regulation of Pellino2 as part of TLR9 signaling. We generated BMDCs from transgenic *Peli2*-tagged mice (Supplemental Fig. 1) to monitor protein levels of Pellino2 during time-dependent stimulation by CpG. Interestingly, the levels of Pellino2 decreased with increasing times after CpG stimulation (Fig. 6A). Given the role of Pellino2 in mediating the proinflammatory response in BMDCs to CpG, its levels may be subject to tight regulation to provide a self-regulatory mechanism for TLR9 to terminate its own proinflammatory response. To assess if Pellino2 may be a limiting factor in TLR9 signaling, we examined whether forced overexpression of exogenous Pellino2 would impact on TLR9-induced cytokine production by DCs. Exogenous Pellino2 was expressed at high levels using a lentivirus system. Overexpression of Pellino2 alone was not sufficient to drive cytokine expression but notably greatly augmented CpG-induced expression of IFN-β and IL-12p70 (Fig. 6B). Next, given the low levels of Pellino2 in BMDMs, we were keen to explore if Pellino2 is a limiting factor in precluding macrophage production of type I IFNs. It has been previously reported that BMDMs do not express type I IFNs in response to CpG (40). We thus explored whether forced overexpression of exogenous Pellino2 would impact on the macrophage response to TLR9 stimulation. However, although the TLR3 ligand poly I:C induced IFN-β secretion in BMDMs, these cells failed to produce IFN-β in response to CpG, and overexpression of Pellino2 did not force BMDMs to produce IFN-β (Fig. 6C). These data suggest that Pellino2 may be a key limiting factor in the TLR9 pathway, leading to cytokine production in a DC-specific manner.

Pellino2 has two functional domains, namely FHA and RING-like domains, that mediate its activity. It has been shown in previous reports that the FHA domain mediates substrate recognitions (41, 42), whereas the RING-like domain confers E3 ubiquitin ligase activity of Pellino2 (43–45). Having provided evidence for a role for Pellino2 in TLR9 signaling in DCs, we focused on the functional relevance of the FHA and RING-like domains of Pellino2 for this role. We used lentivirus transduction to reconstitute *Peli2*^−/−^ BMDCs with cDNA encoding for Pellino2 and mutated forms with loss of function mutations in its FHA or RING-like domains (Fig. 6D). As BMDCs are not transduced in 100% when using lentivirus system, we sorted GFP^+^ cells and stimulated them with CpG. The reintroduction of Pellino2 into *Peli2*^−/−^ BMDCs significantly upregulated induction of IFN-β and IL-12p70. However, mutated forms of Pellino2, the FHA mutant, and RING mutant forms failed to increase levels of cytokines in *Peli2*^−/−^ BMDCs (Fig. 6D). These data indicate that Pellino2 is dependent on both domains and supports essential roles for its substrate binding domain and its E3 ubiquitin ligase activity in manifesting this critical regulatory role in the TLR9 signaling pathway.

### Pellino2 contributes to TLR9 response to *S. aureus* in DCs

**FIGURE 7.**
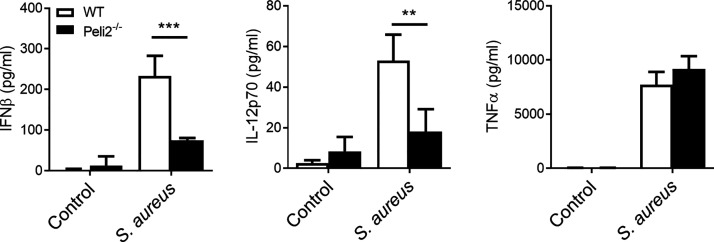
Pellino2 mediate innate response to *S. aureus* in DCs. ELISA of IFN-β, IL-12p70, and TNF-α secreted in medium from WT and *Peli2*^−/−^ BMDCs treated with PBS as control or infected with *S. aureus* (multiplicity of infection [MOI] 50:1). Results show representative data from two independent experiments. Data show means + SD. WT BMDC versus *Peli2*^−/−^ BMDC. **p < 0.01, ***p < 0.001, two-way ANOVA.

We next extended our studies to explore the physiological relevance of the role of Pellino2 as a mediator in the TLR9 pathway. It has been reported that TLR9 activation is crucial for type I IFN induction following infection of DCs with the Gram-positive bacteria *S. aureus* infection in DCs (10). We thus compared the responsiveness of BMDCs from WT and *Peli2*^−/−^ mice to *S. aureus*. In this study, we show that *S. aureus* infection induced production of IFN-β, IL-12p70, and TNF-α in WT BMDCs. However, Pellino2-decificient BMDCs secreted reduced levels of IFN-β and IL-12p70 in response to infection by *S. aureus* relative to WT cells, whereas expression levels of TNF-α levels were not affected (Fig. 7). These data confirm the physiological relevance of Pellino2 as a mediator in the TLR9 pathway in DCs with a specific role in regulating IFN-β and IL-12p70 expression.

### Pellino2 mediates downstream T cell polarization into Th1 subset following TLR9 activation in DCs

**FIGURE 8.**
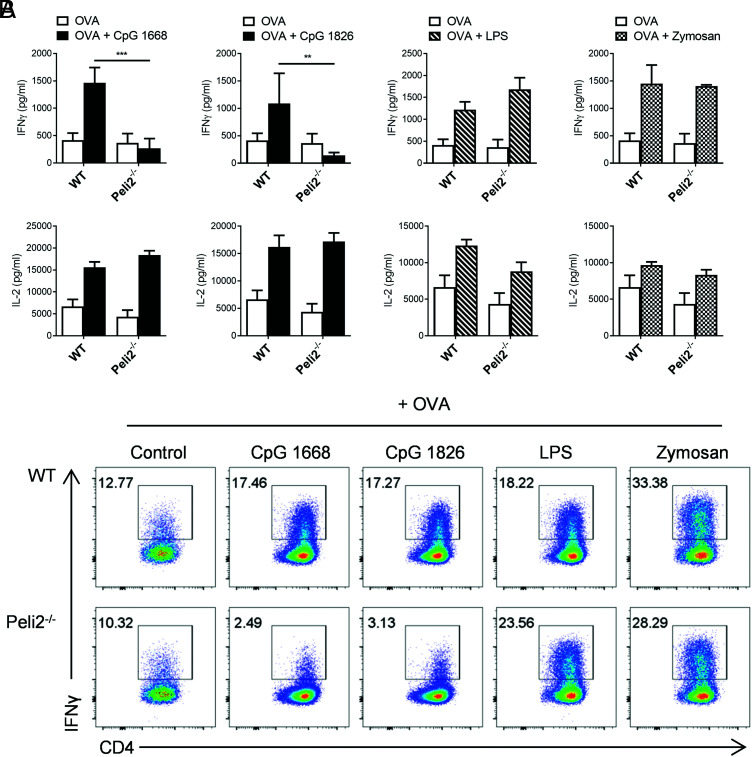
Pellino2 expression in DCs is critical for Th1 polarization of naive CD4 T cells. (**A**) ELISA of IFN-γ and IL-2 from coculture of WT or *Peli2*^−/−^ BMDCs treated with OVA alone or OVA with 1 µg/ml CpG ODN 1668 and 1826, 5 ng/ml LPS, or 1 µg/ml zymosan with OT-II cells (ratio 1:5). (**B**) Intracellular staining of IFN-γ^+^ OT-II cells cultured as in (A). Results are representative of three (A) and two (B) independent experiments. WT OVA + CpG versus Peli2^−/−^ OVA + CpG. **p < 0.01, ***p < 0.001, two-way ANOVA.

Having demonstrated a key role for Pellino2 in mediating TLR-induced expression of proinflammatory cytokines, we were keen to investigate the downstream functional consequences of this effect. IL12p70 is a key polarizing cytokine that promotes differentiation of naive CD4^+^ T cells into Th1 subpopulation, which produce IFN-γ (16). Therefore, as Pellino2-deficient BMDCs produce less IL-12p70 than WT BMDCs, we investigated the ability of these cells to induce differentiation of naive T cells into Th1 cells. WT and Pellino2-deficient BMDCs were stimulated with the Ag OVA and different TLR ligands, such as two types of CpG ODN 1668 and 1826, LPS, or zymosan. Next, naive CD4^+^ T cells from OT-II mice that express a transgenic TCR for OVA were coincubated with the stimulated WT and Pellino2-deficient BMDCs. As expected, WT BMDCs treated with TLR ligands (CpG, LPS, or zymosan) with OVA induced increased IFN-γ and IL-2 production by T cells (Fig. 8A). These responses were still apparent with Pellino2-deficient BMDCs costimulated with OVA and LPS or zymosan. However, Pellino2-deficient BMDCs costimulated with either form of CpG and OVA failed to upregulate IFN-γ secretion, whereas IL-2 production was intact (Fig. 8A). To confirm that CD4^+^ OT-II T cells were the source of IFN-γ, cultured cells were analyzed for surface CD4 and intracellular IFN-γ expression (Fig. 8B). Again, WT and Pellino2-deficient BMDCs treated with OVA and LPS or zymosan were able to induce IFN-γ production, and CD4 T cells were the source of this cytokine. In contrast, Pellino2-deficient BMDCs costimulated with CpG and OVA failed to upregulate IFN-γ secretion in CD4 T cells. These results confirm that Pellino2-deficient BMDCs produce lower levels of bioactive IL-12p70, resulting in impaired differentiation of Th1 T cells.

## Discussion

PTMs including ubiquitination are crucial in regulation of immune responses (19). It is emerging from various reports that DC activation and maturation and cytokine secretion can be regulated by these PTMs. For instance, MHC II levels are regulated by the E3 ubiquitin ligases MARCH1 (46), Hrd1 (47), and WWP2 (48), whereas CD86 upregulation is dependent on MARCH1 (49, 50). IL-6 is regulated by deubiquitinase Rhbdd3 (51), whereas IL-12p40 is controlled by Trabid (52). IL-23 expression is regulated by E3 ubiquitin ligase DCAF2 (53) and deubiquitinase Trabid (52). Our report on Pellino2 adds more mechanistic insight to our understanding of DC functions and how they are regulated by the ubiquitination pathway. In this study, we identify a (to our knowledge) novel role for the E3 ubiquitin ligase Pellino2 in the TLR9 signaling pathway. The findings highlight a cell-specific role for Pellino2 in DCs in regulating proinflammatory cytokines such as type I IFN, IL-12, and IL-6 levels and subsequent T cell differentiation into Th1 subpopulation. We also show for the first time, to our knowledge, that Pellino2 may be a key limiting factor in controlling the level of inflammation. Although Pellino2 is crucial to fully license the cells to respond to TLR9 activation, its protein levels decrease over time in response to TLR stimulation. This regulatory mechanism to control Pellino2 expression during TLR9 activation is likely to be necessary to maintain sufficient immune response to infection but, at the same time, limit damaging inflammation and autoimmunity.

Pellino proteins play several important and nonredundant roles in various cells in the immune system. Pellino1 regulates various pathways in cells of different origin, such as macrophages (25, 54, 55), microglia (56, 57), B cells (28), T cells (26, 27, 58), and epithelial cells of airways (59), whereas the role of Pellino2 and Pellino3 has been mostly characterized in macrophages (29–33). In this study, for the first time, to our knowledge, we show that Pellino2 plays a cell-specific role in DCs. Pellino2 is essential for TLR9 activation and regulates IL-6, IL-12p40, and IL-12p70 production and corresponding gene expression in DCs. TLR activation leads not only to cytokine production but also to DC maturation. However, it appears that the functional role of Pellino2 in DCs does not extend to the maturation pathway and is instead limited to controlling the expression of particular cytokines. The IL-12 family of cytokines are crucial players in T cell polarization into effector Th1 and Th17 cells (60). IL-12p70, a heterodimeric cytokine composed of two subunits, IL-12p35 and IL-12p40, induces Th1 polarization and IFN-γ production by T cells (16). In this report, we show that Pellino2-mediated IL-12p70 production is crucial to driving IFN-γ–producing T cells following TLR9 activation of DCs, whereas at the same time, IL-2 production is not abrogated. Thus, Pellino2 expression in DCs is not required for activation of T cells but plays an important mediatory role in promoting DC-mediated polarization of Th1 cells. Interestingly, Pellino2 is not required to facilitate Th1 polarization in response to DCs activated by TLR2 or TLR4 ligands. This result further supports our data that Pellino2 has a highly specific role in DCs, as it facilitates production of polarizing signals in response to triggering of the TLR9 pathway.

Previous reports described that Pellino2 activates MAPKs pathway (22) and mediate TLR/IL-1 signaling pathways (61). These results would suggest that Pellino2 would support induction of cytokine production via NF-κB or MAPK pathways in DCs. However, we did not observe any role for Pellino2 in these pathways in DCs stimulated with TLR9 ligand. Previous publications focused on overexpression experiments and cell lines and did not explore the role of endogenous Pellino2 in primary cells. Indeed, in our previous report on the role of Pellino2 in NLRP3 pathway in macrophages, we also did not observe any role for Pellino2 in these signal transduction pathways in primary macrophages (33).

IFNs are produced endogenously by DCs in response to TLR activation and enhance downstream IL-12 production (17). However, we show in this study that IFNAR signaling is partly responsible in mediating synergistic effect of type I IFN on IL-12 production. Furthermore, we show that Pellino2 regulates type I IFN production in DCs following TLR9 activation and *S. aureus* infection. We also confirmed that Pellino2 does not play a direct role in type I IFN/IFNAR signaling pathway. We showed that IFN-induced phosphorylation of STAT1 is intact in WT and Pellino2-deficient DCs and there is no difference in varying IFN-responsive gene expression following IFN-β stimulation in these cells. Additionally, the involvement of Pellino2 in type I IFN regulation appears to be highly specific to TLR9, as it does not regulate IFN-β production following activation of different DNA sensors or their mediator STING in DCs. Together, these data suggest that Pellino2 regulates primary IFN-β production rather than the downstream IFNAR signaling pathway. Interestingly, the role of Pellino2 in the TLR9 pathway appears to be restricted to DCs and does not extend to macrophages. It has previously been shown that macrophages fail to phosphorylate STAT1 at Tyr701 when stimulated with CpG, and these cells do not express IFN-β (40). Although macrophages express relatively low levels of Pellino2, its enhanced expression is not sufficient to bestow on macrophages the ability to induce IFN-β. Thus, various factors underlie the divergent roles of macrophages and DCs in immune responses.

This study shows for the first time, to our knowledge, that Pellino2 regulates DNA sensing. Although roles for Pellino1 and Pellino3 in TLR9 signaling have not been described, they are involved in regulating RNA recognition via TLR3 or during antiviral responses. Pellino1 regulates antiviral immune responses to multiple RNA viruses such as West Nile virus (62), Zika virus (63), rhinovirus (64), and vascular stomatitis virus (56), whereas we have previously delineated a role for Pellino3 in response to encephalomyocarditis virus (29). Pellino1 has been shown to regulate IFN-β production in macrophages and microglia (25, 56), whereas Pellino3 controls type I IFNs in macrophages and DCs following TLR3 activation (29). Overall, our data further highlight that Pellino proteins play various distinct functions in immune signaling pathways.

Interestingly, Pellino2 expression is downregulated following TLR9 activation, which suggests that although Pellino2 positively regulates type I IFN expression, its expression is tightly regulated to limit the level of inflammation. Indeed, when Pellino2 is overexpressed, DCs secrete excessive amount of IFN-β and IL-12p70. Importantly, reintroduction of mutant forms of Pellino2, which lack functional domains, failed to reconstitute TLR9 signaling. This shows that Pellino2 needs its substrate binding activity (mediated by FHA domain) and E3 ubiquitin ligase activity (mediated by RING-like domain) to execute its functions. As Pellino2 expression is critical for the outcome of TLR9 activation, it potentially can be exploited in novel therapies in which Pellino2 levels can be controlled to either downregulate or upregulate DC-mediated inflammation.

## Supplementary Material

Data Supplement
