# Supplementary material for: Dendritic Cell–Specific Role for Pellino2 as a Mediator of TLR9 Signaling Pathway
Source: J Immunol. 2021 Nov 1;207(9):2325–36. doi: 10.4049/jimmunol.2100236 (PMC8525870; doi:10.4049/jimmunol.2100236)
Supplement: Data Supplement [file JI_2100236.zip › JI_2100236_Supplemental_1.pdf]

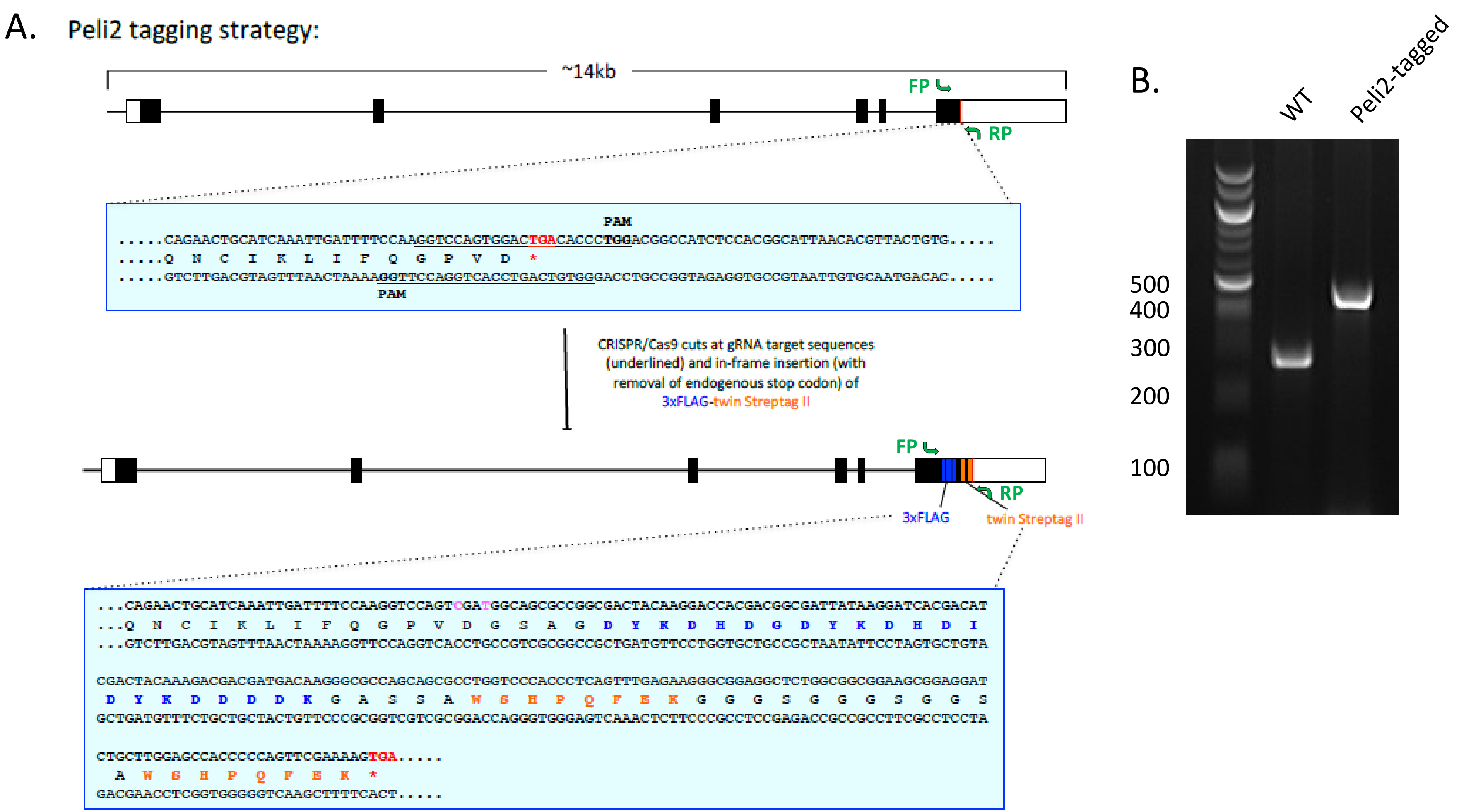

Supplementary Figure 1. CRISPR/Cas9 targeting strategy for the PELI2 FLAG-streptag insertion and genotyping. (A) The mouse PELI2 gene comprises 6 exons (black boxes). Two gRNA target sequences were identified after the end of the coding region in exon 6 (blue box 1, underlined). Upon CRISPR/Cas9 cutting at this site the repair vector provides a template for insertion of a triple FLAG, double Streptag II at the C-terminus *via* homology directed repair (HDR). The repair vector contains two base changes to alter the gRNA target regions to inhibit recutting (blue box 2, pink). (B) Genotyping by PCR analysis of genomic DNA from ear punches. Primers differentiate the wild-type (WT) allele and targeted allele by amplifying different length of DNA (wild-type: 298 bp; tagged band: 493 bp).
